# Prostate Stereotactic Body Radiotherapy With Synchrony®-Based Fiducial Tracking on Radixact® X9

**DOI:** 10.7759/cureus.83742

**Published:** 2025-05-08

**Authors:** Adithya Vikrama Acharya, Sanjay Hunugundmath, Mariya Deputy, Amit Nirhali, Vishram Naik, Sharad Gadhave

**Affiliations:** 1 Radiation Oncology, Sahyadri Superspeciality Hospital, Pune, IND; 2 Radiation Therapy, Sahyadri Superspeciality Hospital, Pune, IND

**Keywords:** extreme hypofractionation, fiducial-based tracking, helical tomotherapy, image-guided radiotherapy, late-reacting tissue, localized prostate cancer, prostate hypofractionation, real-time intrafraction management, reduced treatment time, stereotactic ablative body radiotherapy (sbrt)

## Abstract

Prostate cancer, when detected early, can be either actively observed or treated radically based on clinical, pathological, and imaging features. Radical treatment options include radical prostatectomy, radical radiation as external beam radiotherapy (EBRT), or brachytherapy. EBRT for prostate cancer can be given in different ways. It may be delivered in smaller doses over several weeks, known as conventionally fractionated radiation therapy (CF-RT), or in larger doses over fewer sessions, as in moderately hypofractionated radiotherapy and ultra hypofractionated radiotherapy, also known as stereotactic body radiation therapy (SBRT). Recent studies have shown that SBRT is non-inferior to CF-RT, and the National Comprehensive Cancer Network guidelines also suggest SBRT as a treatment option for localized disease. As SBRT uses a high dose per fraction for treatment, it requires a very tight margin around the planning target volume and intrafractional motion management to prevent the organs at risk from receiving a high dose and subsequent toxicities developing from it. In this case series, we report our clinical experience with prostate SBRT using the Radixact® X9 (Accuray, Sunnyvale, CA, United States) tomotherapy machine with Synchrony® fiducial tracking for real-time motion tracking and correction.

## Introduction

Prostate cancer, according to the Global Cancer Observatory (GLOBOCAN) 2022 data, ranks fourth in incidence and eighth in mortality globally. In cancers with male predominance, it secures second and fifth positions, respectively, in terms of incidence and mortality in male cancers, and with respect to the Indian subcontinent, it ranks 12th and 14th places, respectively, [1]. It can be organ-confined, locally advanced (extension to seminal vesicles, surrounding structures and nodes), or metastatic in nature. Most common presentation is asymptomatic disease with incidental raised prostate-specific antigen (PSA) values on general biochemical workup.

In early stage disease, i.e., organ-confined disease (one or both lobes of the prostate gland), several treatment options are available depending on the clinical, pathological, and imaging findings. The options include active surveillance, radical prostatectomy, and radical radiation as EBRT or brachytherapy (low-dose rate [LDR] radioactive seed implant brachytherapy or high-dose rate [HDR] interstitial brachytherapy). In the 15-year update of the ProtecT trial [2], the prostate cancer-specific mortality was low, regardless of the treatment assigned, and the choice of therapy involved weighing the pros and cons associated with treatments for localized prostate cancer.

Radiation is an exceptionally effective treatment for prostate cancer of any risk group [3]. Historically, conventionally fractionated radiation therapy (CF-RT) i.e., 1.8-2 Gray (Gy) per fraction, five days a week, to a total dose of up to 80 Gy, was given. This protracted the treatment over seven to nine weeks, which impacted the patient’s quality of life and utilization of hospital resources.

For most cancers, the alpha/beta (α/β) ratio is high (10 Gy), indicating that these tissues are more sensitive to total radiation dose rather than dose per fraction, and for the late-reacting surrounding normal tissues, it is low (3 Gy), indicating a higher sensitivity to fraction size. Multiple studies from LDR brachytherapy treatment for prostate cancer suggest that the α/β is possibly as low as 1.5 Gy [4-6], leading to the revelation that the prostate is a late-reacting tissue. This led to the assumption and hypothesis that hypofractionated schedules for prostate cancer would produce tumor control and late treatment-related sequelae that are at least as good or better than those currently achieved with CF-RT.

Need for hypofractionation

To test the hypothesis, multiple phase III randomized controlled trials (RCTs) testing moderate hypofractionation (2.5-4 Gy) against conventional fractionation were conducted [7]. These RCTs proved that moderate hypofractionated radiation therapy (HF-RT) is superior/non-inferior to CF-RT and can be implemented clinically worldwide but at the cost of slightly higher incidence of ≥grade 2 genitourinary toxicity. This led to moderate HF-RT for localized prostate cancer being the current standard of care.

From the RCTs, it is clear that increasing the dose to the prostate increases cure rates at the expense of increased side effects. It may be possible to simultaneously increase cure rates while decreasing the toxicity by exploiting the unusual radiobiology of prostate cancer.

For prostate cancer patients, the dose-limiting structure are the rectum, the urethra, and the urinary bladder as they lie in close proximity to the prostate gland, and their α/β ratio is the same as that of the late-reacting normal tissues, i.e., 3 Gy [6,8].

Taken together, the α/β ratio of prostate cancer appears to be significantly lower than that of the rectum, the urethra, and the urinary bladder, which means that the higher the dose per fraction, the higher the cell kill to the prostate cancer. This should be accompanied by a reduction in the incidence of rectal side effects due to the lower total dose required.

After realizing that moderate HF-RT is as good as or superior to CF-RT, the next question to answer is whether ultra-hypofractionation (>5 Gy per fraction) could produce the same non-inferior results as moderate hypofractionation.

Historically, the delivery of larger fraction sizes is limited by normal tissue constraints and the requirement for large planning margins. Stereotactic body radiation therapy (SBRT) is a highly precise radiation treatment technique that delivers large doses of radiation in less fractions.

Early data from an Accuray (Sunnyvale, CA, United States) sponsored study by Fuller et al. [9] showed that five-fraction SBRT for low-risk prostate cancer is feasible, with a biochemical progression-free survival (bPFS) of 93%, and comparable to HDR brachytherapy in many respects, with a lower incidence of ≥grade 2 genitourinary toxicity.

Cushman et al. [10] conducted a systematic review and meta-analysis of phase II prospective trials of SBRT for prostate cancer and concluded that with the available evidence at that point of time, prostate SBRT can afford appropriate biochemical control of 90-99% with few high-grade toxicities.

A large series of more than 1,000 patients from a multi-institutional consortium of prospective trials [11,12] confirmed that SBRT results in five-year biochemical control rates similar to those seen with conventional fractionation and is associated with only transient declines in quality of life.

Two recent large phase III RCTs, namely HYPO-RT PC [13] and PACE-B [14], have shown the efficacy of prostate SBRT.

SBRT is now considered as a treatment regimen for low- and intermediate-risk clinically localized prostate cancers and has been incorporated in the treatment guidelines of National Comprehensive Cancer Network (NCCN). In the recent years, data emerging from phase II trials such as SATURN, FASTR, and FASTR2 have demonstrated the feasibility of SBRT in high-risk diseases. The NCCN also supports for SBRT in high-risk cases in selective scenarios.

As SBRT utilizes a very tight margin, the management of intrafraction motion of the target volume is considered extremely important. The goal of intrafraction motion management is to safely deliver as much radiation as possible to the tumor while protecting nearby healthy organs, known as organs at risk (OARs), from damage. Intrafraction motion in the prostate can be significant owing to a combination of factors such as muscle tension or relaxation, respiratory motion, bowel movement, and rectal and bladder filling.

Prostate SBRT can be delivered using various types of platforms, such as robotic-arm-mounted linear accelerators (LINAC), i.e., the CyberKnife® (Accuray) system, gantry-mounted LINAC, and magnetic resonance imaging (MRI)-guided LINAC. Only gantry-mounted LINAC is commonly available at treatment centers due to limited resources. Therefore, an effective solution is required to manage intrafraction motion when using gantry-mounted LINAC. Hence, systems to verify positioning and monitoring the movement of organs and anatomical regions of interest were developed. In the current clinical practice, fiducial tracking and daily imaging in the form of X-rays or cone beam scans are being used.

As technology advanced, LINAC became capable of stereotactic therapy via volumetric modulated arc therapy (VMAT). VMAT technique allows the machine to rotate around the patient while continuously adjusting both the shape and intensity of the radiation beam. This is done using small moving parts called multileaf collimators (MLCs), which help target the tumor more precisely and reduce radiation to healthy tissue. These enable a dosage distribution that is exceptionally compliant to the target volume while conserving more normal tissue. With the introduction of systems for verifying positioning and monitoring the movement of organs and anatomical regions of interest, enhanced safety can be attained in the delivery of ultra-hypofractionated regimens, and true stereotactic treatments can be created using the VMAT technique.

In a study by Serra et al. [15], treatment plans for 17 prostate cancer patients were compared across three SBRT methods: CyberKnife®, VMAT, and helical tomotherapy (HT). The aim was to see how each approach performed in terms of how the dose was delivered and how well healthy tissue was spared. The study revealed that although CyberKnife® is the best SBRT technique, it can be replaced by a similar treatment delivered by VMAT or HT technique with an appropriate image-guided radiation therapy (IGRT) tracking system.

The purpose of this study was to test the feasibility of Synchrony® fiducial tracking system on the Radixact® (Accuray) tomotherapy machine using HT plans.

At our center, the Radixact® X9 tomotherapy was commissioned in July 2024, and until now, seven patients have been treated with SBRT using Synchrony® fiducial tracking system to a dose of 36.25 Gy in five consecutive fractions. The inclusion criterion was males >18 years, with proven organ-confined disease, no nodal involvement, no distant metastasis (cT1-T2, N0, M0) on imaging (MRI or positron emission tomography), histopathologically proven adenocarcinoma of the prostate, and serum PSA levels of 10-20 ng/mL.

## Materials and methods

This case series was conducted at a tertiary care hospital at Pune (a metro city in the state of Maharashtra, India) with a full-fledged oncology setup. Clinical and imaging data were obtained from both electronic and physical records. The criteria for inclusion were males >18 years, with proven organ-confined disease (cT1/cT2) on imaging (MRI or positron emission tomography), histopathologically proven prostate cancer, and serum PSA levels of 10-20 ng/mL. Exclusion criteria were advanced disease (cT3/cT4), involvement of regional lymph nodes (cN+), and oligometastatic or full-blown metastasis at presentation. Seven such patients were identified and classified according to the NCCN prostate cancer risk stratification.

Prior to radiation treatment, the patients underwent radio-opaque fiducial placement under ultrasound guidance by an interventional radiologist. Three radio-opaque fiducials were placed in the prostate after taking all aseptic precautions. Gold fiducials are most commonly used as they have a high atomic number (Z) value and, as a result, are highly visible with X-ray imaging. Also, gold is relatively inert, is easy to deliver with good visibility, has minimal distortion on CT imaging, has minimal dose perturbation, is biocompatible with soft tissue, and has a negligible risk of migration. The fiducials were placed in such a way that each of them was in a different plane and a few centimeters apart with respect to each other such that they were clearly visible for verification during treatment delivery.

One to two weeks after fiducial implantation, the patient was asked to come for the radiation planning simulation. The patient was first asked to evacuate both the bowel and the bladder and was immobilized using a vacuum lock (VacLok) bag. The immobilization was done in such a way that the patient was comfortable and snugly fit into the VacLok. No other immobilization devices were used. Then, 400 mL of water was given to the patient as bladder protocol (institutional protocol). After 30-40 minutes or depending on the patient’s bladder capacity, a non-contrast CT scan of the pelvis with slice thickness of 1 mm was performed using the SOMATOM Go NOW (Siemens Healthineers, Erlangen, Germany) 16-slice CT scan machine. During the simulation, the patient was asked to do a mid-breath hold to have a clear visibility of all the fiducials and to reduce the movement artifacts seen during respiration. If the bladder was not adequately filled or if there was gas in the rectum (>3-4 cm in diameter), the patient was simulated again until the bladder was adequately filled and/or the rectum was completely collapsed. The scans were taken in all sections (axial, coronal, and sagittal). For co-registration and accurate delineation of the prostate, urethra, and penile bulb, a contrast-enhanced MRI of the pelvis was performed using a SOMATOM ESSENZA (Siemens Healthineers) 1.5-Tesla MRI machine. Both imaging scans were performed either on the same visit or within a few days of each other.

The MRI images were co-registered along with the treatment simulation CT scan using the rigid registration option available in the treatment planning software/system (TPS). The OARs and the target volume were delineated in each and every slice. For accurate delineation of the prostate, seminal vesicles, urethra, and penile bulb, MRI was used. The dose prescribed to all cases was 36.25 Gy in five fractions on consecutive days. The plans after evaluation and approval were executed on Radixact® X9 tomotherapy machine with real-time fiducial tracking using Synchrony®.

Following treatment, the patients had minimal acute toxicities and are regularly followed up to date.

## Results

Case 1

A 71-year-old man with a history of type 2 diabetes mellitus and hypertension presented with urinary retention lasting 15 days in April 2024. A serum PSA measured in July 2024 was elevated at 14.23 ng/mL. A contrast-enhanced MRI of the pelvis performed in May 2024 revealed an enlarged prostate measuring 44x37x37 mm (volume 30 cc), with a PSA density of 0.39 ng/mL². A nodule in the mid-gland peripheral zone on the left side, measuring 14x12 mm, showed diffusion restriction, T2 hypointensity, and early enhancement-features consistent with a PIRADS (Prostate Imaging-Reporting and Data System) 4 lesion. Additional PIRADS 3 nodules were noted in the peripheral zone. There was no extracapsular extension, and seminal vesicles, vas deferens, lymph nodes, and bones were normal. A six-core TRUS-guided biopsy revealed prostatic adenocarcinoma (Gleason score 4+5=9, grade group 5), with 50% tumor involvement and perineural invasion (PNI) in all cores. The patient underwent cystoscopy with channel TURP and bilateral orchidectomy in June 2024. Histopathology confirmed the biopsy findings. A technetium 99m methylene diphosphonate (Tc 99m-MDP) bone scan in June 2024 showed no skeletal metastases. He was classified as high-risk localized prostate cancer (cT2bN0M0) and started on neoadjuvant hormonal therapy (NAHT), followed by SBRT to a dose of 36.25 Gy in five fractions. Post-radiation, he was placed on long-term androgen deprivation therapy (ADT). His serum PSA showed a steady decline from 1.63 ng/mL to 0.13 ng/mL.

Case 2

A 74-year-old man without comorbidities presented with increased urinary frequency in May 2024. His serum PSA in August 2024 was 12.81 ng/mL. A multiparametric MRI in August 2024 revealed a 27 cc prostate with a PIRADS 4 lesion in the right peripheral zone, showing T2 hypointensity, mild diffusion restriction with apparent diffusion coefficient (ADC) drop, and increased choline peak on MR spectroscopy. No extracapsular extension or involvement of adjacent structures was noted. A TRUS-guided six-core biopsy from the right lobe showed acinar adenocarcinoma (Gleason score 3+4=7, grade group 2), with 71-80% tumor involvement and PNI. High-grade PIN was also present. Gallium-68 prostate-specific membrane antigen (Ga-68 PSMA) positron emission tomography-computed tomography (PET-CT) demonstrated increased uptake in the entire prostate (SUV 6.53 right, 5.94 left), with faint uptake in bilateral external iliac nodes (SUV 1.45), and no bone or distant metastasis. The patient was staged as unfavorable intermediate-risk localized disease (cT2cN0M0). He underwent short-term NAHT followed by SBRT (36.25 Gy in five fractions), with a post-treatment PSA decline from 1.1 ng/mL to 0.3 ng/mL.

Case 3

A 76-year-old man with type 2 diabetes and ischemic heart disease was initially evaluated for lower back pain and was diagnosed with L3 vertebral compression fracture. FDG PET-CT in February 2023 favored an osteoporotic over metastatic cause, and he underwent spinal decompression and instrumentation in March 2024. Subsequently, he developed urinary symptoms and had a PSA of 12.7 ng/mL in July 2024. TRUS biopsy performed in August 2024 from the right lobe confirmed adenocarcinoma (Gleason score 4+3=7, grade group 3), with 60% tumor involvement. Ga-68 PSMA PET-CT showed focal uptake in the right peripheral zone (SUV 8.9) with no nodal or skeletal metastases. MRI of the prostate in December 2024 showed a 38 cc prostate with an ill-defined hypointense lesion in the right peripheral zone. No extracapsular extension or seminal vesicle involvement was present. He was classified as unfavorable intermediate-risk localized disease (cT2bN0M0) and treated with NAHT followed by SBRT (36.25 Gy in five fractions). His PSA declined to 0.1 ng/mL post-treatment.

Case 4

A 66-year-old man with type 2 diabetes and ischemic heart disease post-PTCA presented with dribbling and burning micturition. Ultrasound in March 2024 revealed moderate prostatomegaly and significant post-void residual. PSA in May 2024 was 10.37 ng/mL. Following cystoscopy, internal urethrotomy, and bladder neck incision in June 2024, histopathology confirmed acinar adenocarcinoma (Gleason score 3+4=7, grade group 2), with 15% tumor involvement and no PNI. Ga-68 PSMA PET-CT showed an enlarged prostate (31x30 mm) with PSMA uptake (SUV 4.6) and weak uptake in bilateral external iliac nodes. No distant metastases were detected. He was diagnosed with favorable intermediate-risk localized disease (cT2bN0M0) and treated with SBRT (36.25 Gy in five fractions), with a post-treatment PSA of 0.1 ng/mL.

Case 5

A 74-year-old man with no known comorbidities was evaluated for lower urinary tract symptoms (LUTS) in May 2024. Ultrasound revealed a mildly enlarged prostate (volume 36 cc), pre-void urine of 260 cc, and post-void residual of 25 cc. PSA was 15.5 ng/mL. A 12-core TRUS-guided biopsy revealed adenocarcinoma in the right lobe (Gleason score 3+3=6, grade group 1) with 60% involvement, no PNI, LVSI, or PIN. The left lobe was benign. Ga-68 PSMA PET-CT showed PSMA-avid lesions in both lobes with SUV max of 12.14 (right) and 18.38 (left), with small external iliac and obturator nodes showing minimal uptake. He was staged as favorable intermediate-risk (cT2cN0M0) and received SBRT (36.25 Gy in five fractions). Post-treatment PSA was 0.21 ng/mL, and follow-up PSMA PET-CT in September/October 2024 showed complete resolution of tracer uptake in the prostate and nodes.

Case 6

A 78-year-old man with type 2 diabetes and hypertension was evaluated for urinary dribbling for 15 days in June 2024. His PSA was 10.5 ng/mL. A six-core biopsy from the right lobe in August 2024 confirmed adenocarcinoma (Gleason score 3+4=7, grade group 2), with 60% tumor involvement. Ga-68 PSMA PET-CT showed an enlarged prostate (4.7x4.5x4.8 cm) with diffuse PSMA uptake (SUV 6.6), and bilateral external iliac nodes without significant activity. No metastases were found. He received a single dose of leuprolide 22.5 mg subcutaneously (sc) as NAHT, followed by SBRT (36.25 Gy in five fractions). Post-treatment PSA dropped to 0.3 ng/mL.

Case 7

A 78-year-old man with type 2 diabetes and hypertension presented with nocturia, inability to retract the prepuce, and right flank pain in March 2024. PSA in April 2024 was 15.7 ng/mL. He underwent cystoscopy, laser optical internal urethrotomy (OIU), urethral dilatation, 11-core TRUS-guided biopsy, circumcision, frenuloplasty, and meatotomy. Intraoperatively, a trilobular prostate and urethral stricture were noted. Histopathology revealed adenocarcinoma (Gleason score 4+3=7, grade group 3), without PNI or lymphovascular space invasion (LVSI). Ga-68 PSMA PET-CT showed an enlarged prostate (4.6x5.1x6.0 cm) with intense tracer uptake (SUV 11.41), but no extra-prostatic involvement. He was classified as unfavorable intermediate-risk localized disease (cT2cN0M0), started on degarelix (loading dose 240 mg followed by 80 mg every 28 days), and later treated with SBRT (36.25 Gy in five fractions). His PSA declined from 1.63 ng/mL to 0.13 ng/mL after treatment.

The clinical characteristics are summarized in Table 1.

**Table 1:** Patient and tumor characteristics c, clinical stage; T, tumor stage; N, nodal stage; M, metastasis; Gy, gray (SI unit for absorbed dose of ionizing radiation)

Patient and tumor characteristics	Number
Total patients	7
Age (years), median (range)	74 (66–78)
T stage	
cT1	0
cT2	7
cT3	0
cT4	0
N stage	
cN1	0
cN2	0
cN3	0
M stage	
M1	0
Risk	
Low	0
Intermediate	6
High	1
Intermediate risk	
Unfavorable	4
Favorable	2
Prostate volume (cc), median (range)	65 (49–80)

Treatment planning and delivery

**Figure 1:**
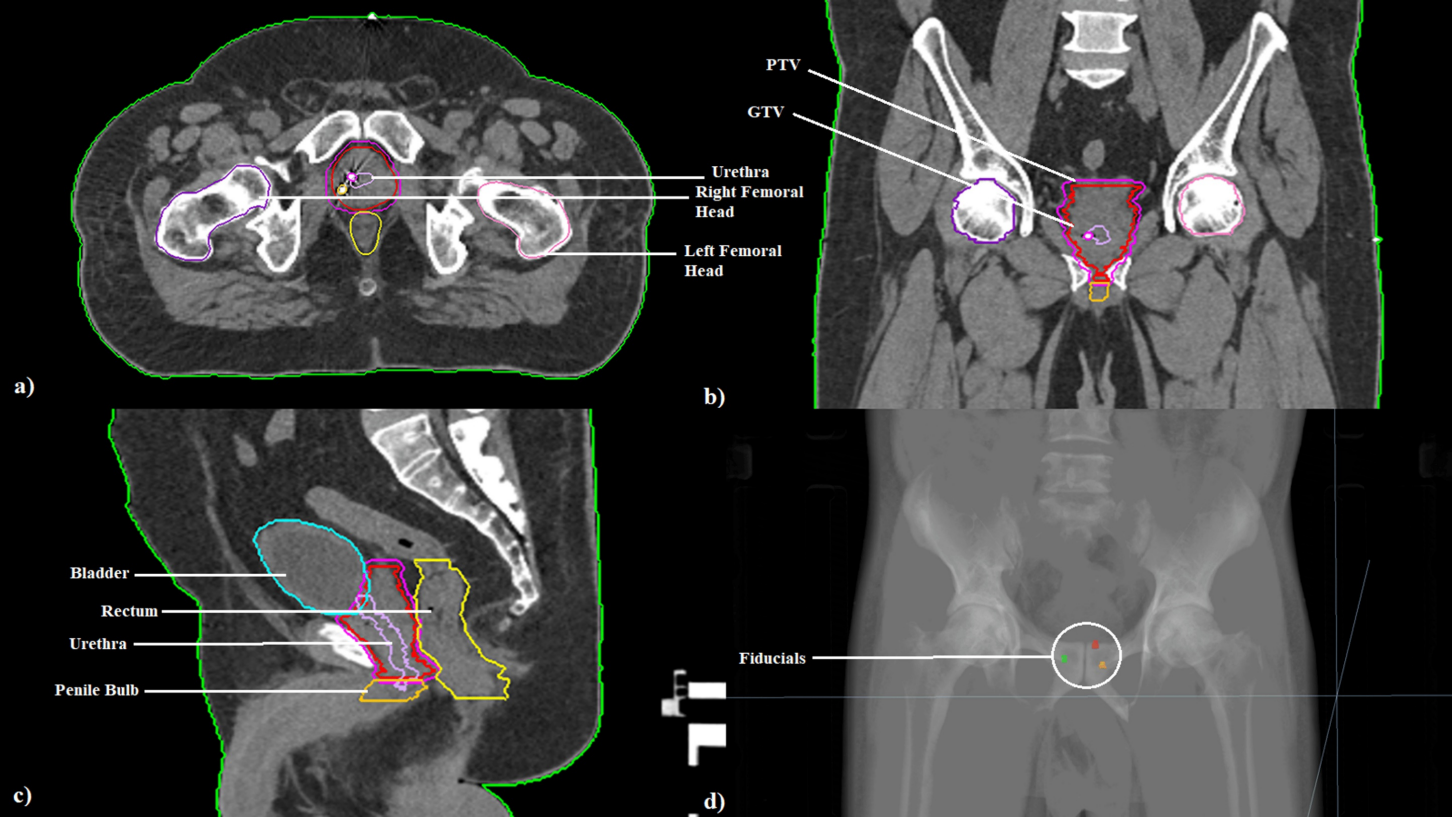
Image depicting target volume delineation in (a) axial, (b) coronal, and (c) sagittal sections with (d) fiducials, as seen in the digital reconstruction GTV, gross tumor volume; PTV, planning target volume

Following radiation planning CT simulation, the Accuray Precision® MD Suite TPS package was utilized to import the CT and MRI images in DICOM (Digital Imaging and Communications in Medicine) format. Target volume delineation was subsequently completed. Both the imaging modalities were fused together using the rigid registration option available in the TPS. The OARs (urinary bladder, rectum, right and left femoral heads, urethra, and penile bulb) were delineated. For better delineation of the urethra and penile bulb, MRI was used. The fiducials were contoured and assigned as tracking markers for Synchrony® (Figure 1).

The gross tumor volume (GTV) is the whole prostate gland and is contoured using the MRI. The clinical target volume (CTV) included the proximal 1 cm of proximal seminal vesicles along with the prostate. The target delineation is done on every slice. An isotropic margin of three millimeters is given as planning target volume (institutional protocol).

**Figure 2:**
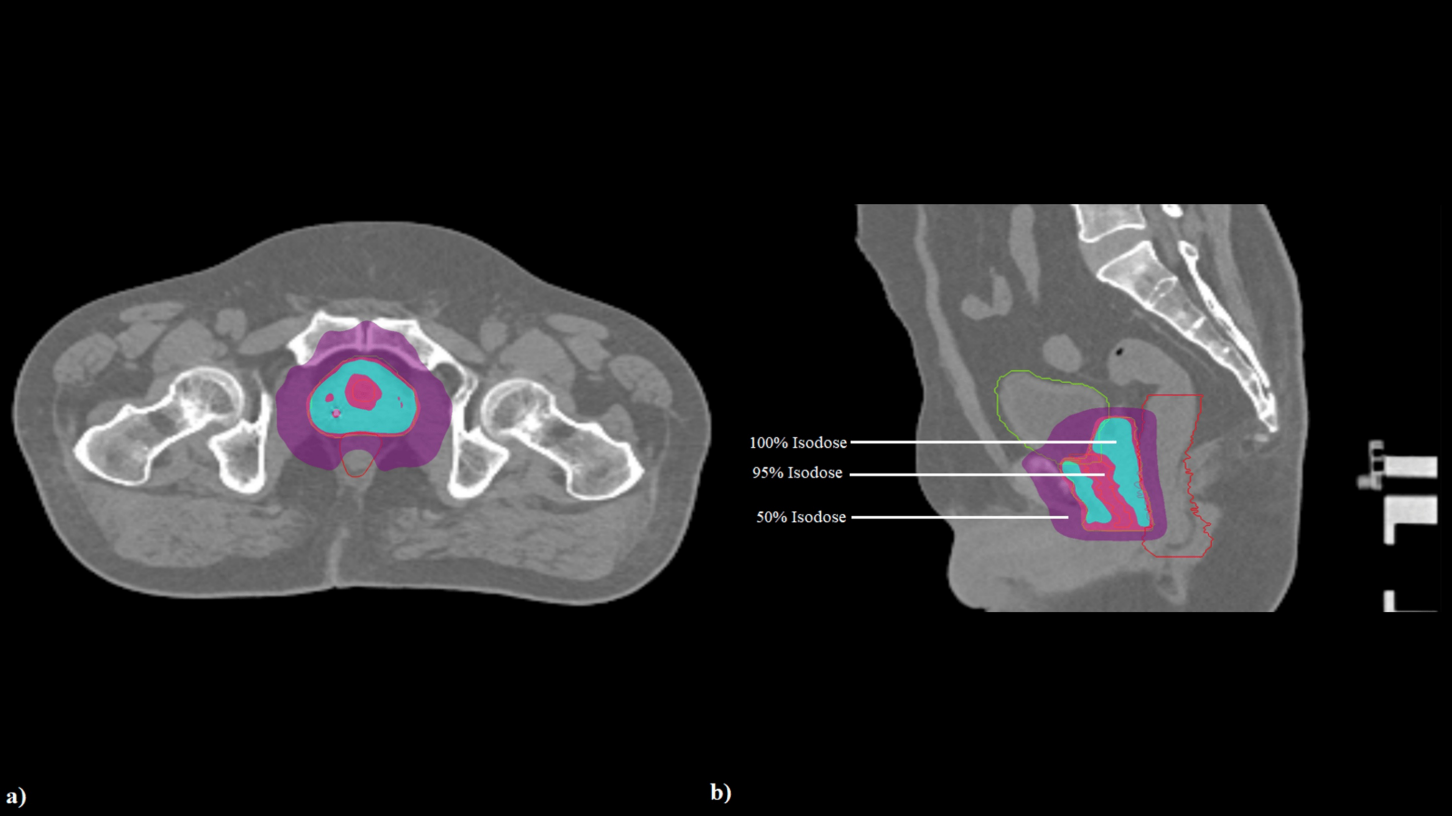
Image showing helical tomotherapy plan in (a) axial section and (b) sagittal section Blue color wash: volume receiving 100% of prescribed dose; magenta color wash: volume receiving 95% of prescribed dose; purple color wash: volume receiving 50% of prescribed dose.

Once target delineation is conducted, planning is done on Accuray Precision® treatment planning system using a field width of 2.5 mm using a 2.5-cm dynamic jaw for high-resolution dose conformity. Moderate modulation is done to optimize dose distribution and treatment time. TomoHelical planning with jaw and MLC optimization is done for motion-adaptive tracking (Figure 2). The Synchrony® model is enabled to track fiducials. VOLO™ Ultra Optimization is used to reduce planning time while maintaining high plan quality and OAR sparing.

The constraints were given as per the ongoing NRG-GU005 trial [16] with minor modifications (Table 2).

**Table 2:** Dose constraints Vx is the volume of the region receiving at least x% of the prescribed dose.

Organ at Risk	Constraints
Rectum	V18.2 Gy < 50%
	V32.63 Gy < 10%
Bladder	V18.2 Gy < 50%
	V38.06 Gy < 0.03 cc
Urethra	V38.78 Gy < 0.03 cc
	V19.9 Gy < 10 cc
Penile bulb	V30 Gy < 3 cc

The constraints achieved for all plans are mentioned in Table 3.

**Table 3:** Dose constraints achieved for all plans Vx is the volume of the region receiving at least x% of the prescribed dose.

Organ at Risk	Constraints	Achieved (in Gy), median (range)
Rectum	V18.2 Gy < 50%	18.1 Gy (18–18.3 Gy)
	V32.63 Gy < 10%	3.63 Gy (3.63–3.65 Gy)
Bladder	V18.2 Gy < 50%	18.1 Gy (18–18.3 Gy)
	V38.06 Gy <0.03 cc	37.25 Gy (36.98–38.3 Gy)
Urethra	V38.78 Gy < 0.03 cc	36.93 Gy (35.96–38.87 Gy)
	V19.9 Gy < 10 cc	10.93 Gy (9.35–16 Gy)
Penile bulb	V30 Gy < 3 cc	20.86 Gy (14.6–29.88 Gy)

After achieving the constraints, the plans were evaluated and approved. After plan approval, the treatment was delivered continuously for five days at a dose of 7.25 Gy per day, using 6 megavoltage (MV) energy on Radixact® X9 tomotherapy machine. The average beam on time was 8 minutes.

Synchrony® setup

Pretreatment Imaging

**Figure 3:**
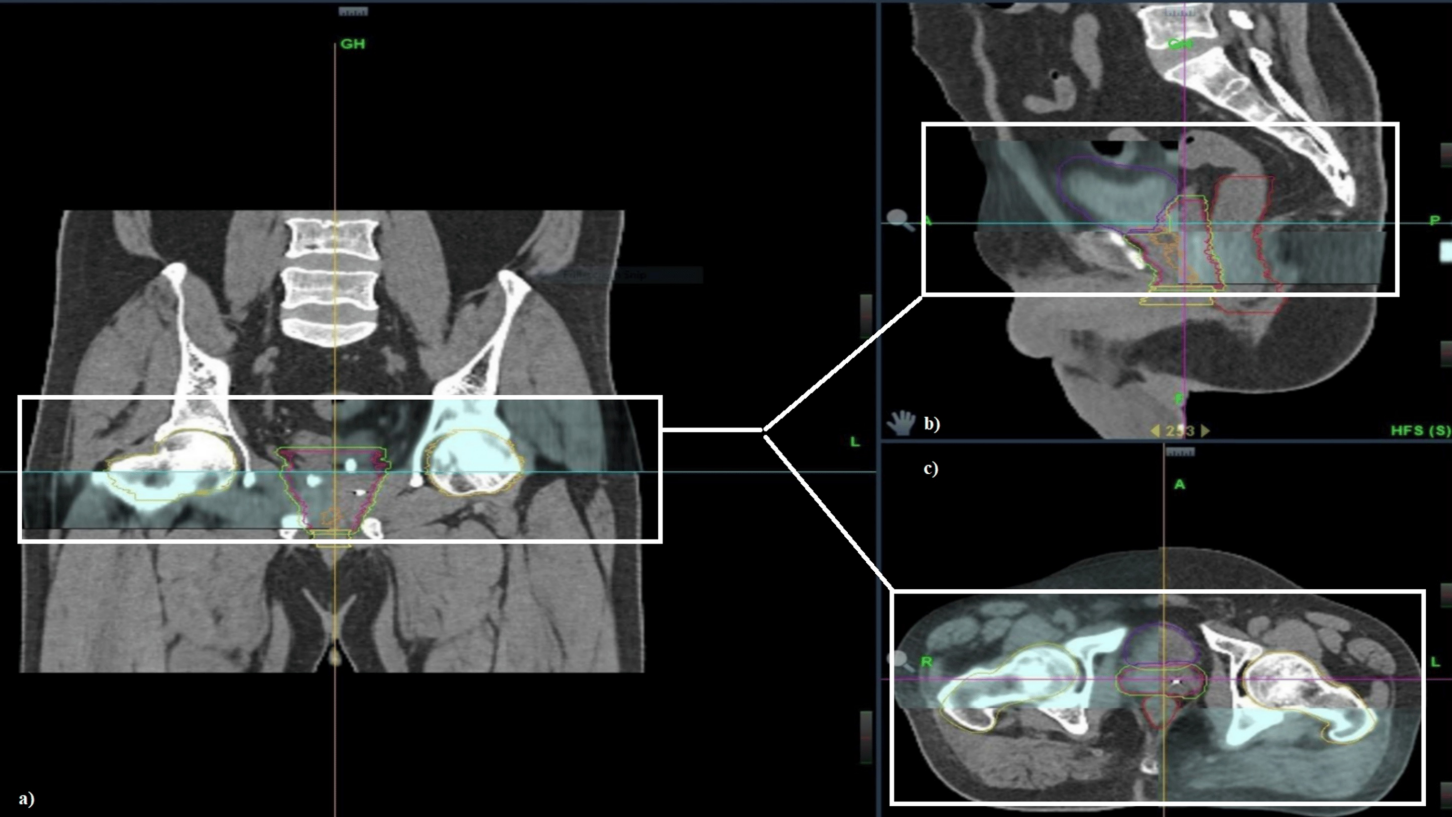
Overlap of daily kV CBCT (white box) imaging with initial simulation scans for verification of position in (a) coronal, (b) sagittal, and (c) axial sections kV kilovoltage; CBCT, cone beam computed tomography

During the treatment, patient positioning was performed using surface-guided radiation therapy (SGRT) system by C-RAD (Uppsala, Sweden), installed as an add-on package. SGRT-based patient positioning enables tattooless and precise positioning. Prior to delivery of each fraction, ClearRT™-based kilovoltage CT (kVCT) was taken to ensure the prostate position and visual verification of the fiducial (Figure 3). This was used to align the prostate or fiducials with the planning CT. Setup corrections if any, were done based on fiducials, not the bony anatomy. Optical markers were placed on the patient's lower abdomen to serve as an external surrogate for motion.

Model Building

A series of orthogonal kV images are taken to detect the fiducial position and create a correlation between the external surrogate and the internal fiducial motion. A minimum of two orthogonal views where the fiducials are clearly visible are required to build the model.

I*maging and Tracking*

Post-position verification, four to six two-dimensional (2D) kV radiographs are taken at standard angles of 0°, 45°, 135°, 180°, 240°, and 315° to create a model for the fiducial localization and for verification of the target position. Additional kV images are taken at other angles as and when required (usually by some degrees) according to the model visibility.

When the radiation beam is toggled on, the position of the fiducials are tracked in real time by a series of 2D radiographs. The model is updated continuously after each gantry rotation (12-20 seconds, depending on the plan) or after a minimum of four radiographs in order to enable real-time treatment adaptation to the target's new position.

Synchrony® model parameters

The synchrony® model parameters are as follows:

Potential Diff (2 mm): this parameter provides a statistical prediction of the 3D distance error that could occur when the current model is used.

Measured Δ (2 mm): this parameter indicates the difference between the predicted and measured target position, which helps assess the model's accuracy.

Rigid Body (2 mm): this parameter reflects the maximum distance difference between fiducial markers in the planning images and the live images, indicating how well the fiducials are matching up.

Thresholds and Auto-Pause (10 sec): the Synchrony® system is configured to pause treatment if any of the model parameters exceed predefined thresholds, ensuring that the treatment is not delivered when the tracking accuracy is compromised.

The thresholds for potential difference, rigid body, and target offset were kept the same for all patients.

Treatment delivery

**Figure 4:**
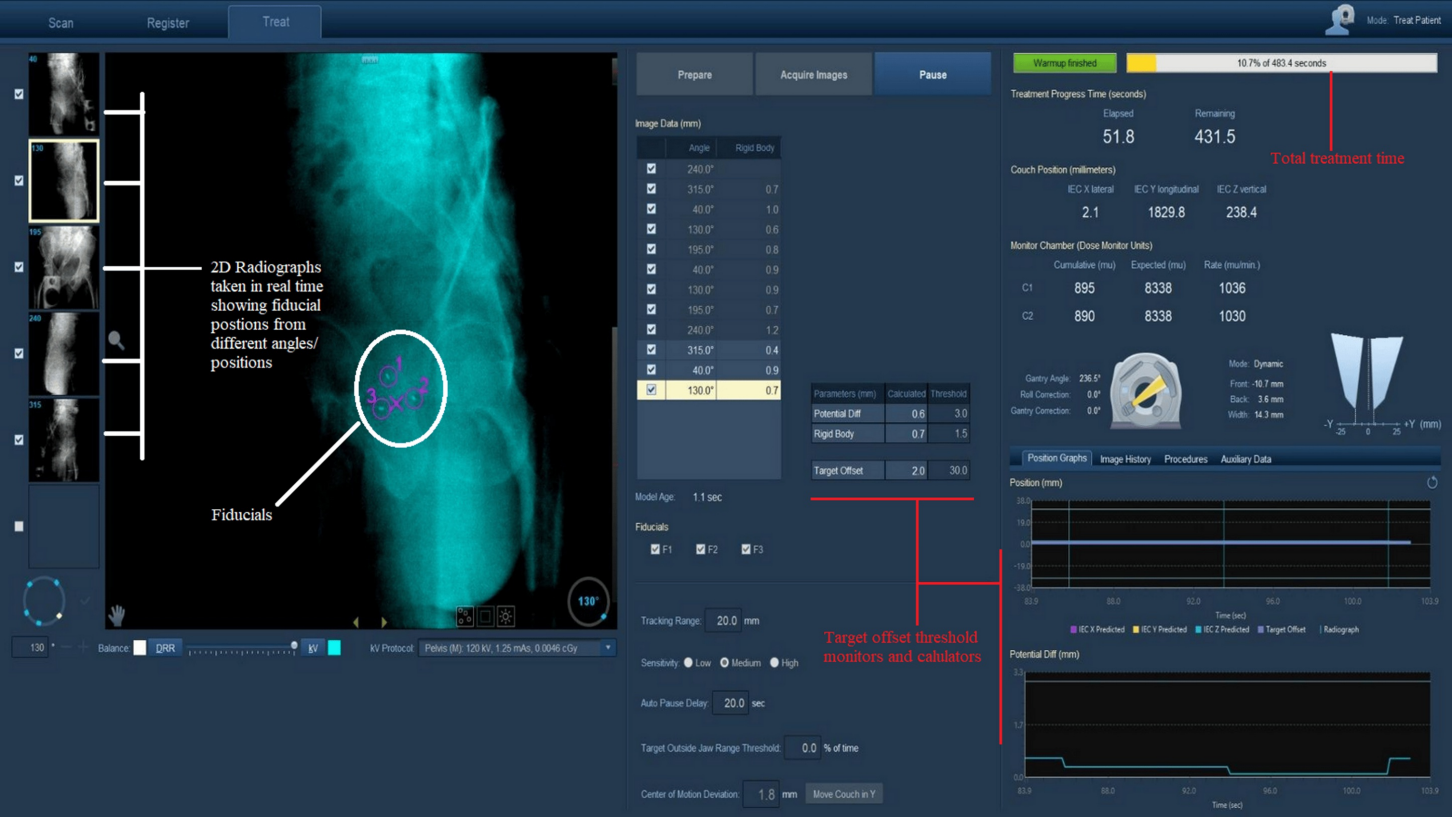
Intra-fraction motion management using Synchrony® for tracking the fiducial position using 2D radiographs from multiple positions continuously in real time

The TomoHelical plan with Synchrony® tracking is enabled. Both the MLCs and jaw adaptation are dynamically adjusted in real time to track tumor motion in all three dimensions (Figure 4). The beam is adjusted or paused if the patient moves, the model error exceeds tolerance, or there is respiratory baseline drift. The treatment is interrupted if the model fails completely or there is excessive patient movement or cough. A new model is built mid-treatment after a quick verification CBCT or kV radiographs, and the treatment is resumed.

## Discussion

Radiation has acquired prominence among clinical treatments for treating early prostate cancer, mostly as an alternative to surgery. Because the prostate has a low α/β ratio, hypofractionated regimens can deliver substantial radiation doses in smaller fractions. Both moderately hypofractionated and ultra-hypofractionated regimens are not inferior to one another.

Taking advantage of the different intrinsic radiosensitivities of the prostate when compared to the surrounding healthy tissues, multiple RCTs conducted on the feasibility, efficacy, dose escalation, and so on, using ultra-hypofractionation demonstrate that SBRT for prostate-confined disease should be offered as a treatment option. SBRT also promised a higher quality of life than surgery by minimizing risks, side effects, and potential complications.

Macias et al. [17] conducted a phase II RCT to assess early urinary and rectal toxicities in patients undergoing SBRT of eight fractions on alternate days using HT without fiducial implants and megavoltage CT images (MVCT) daily for image guidance and showed that acute toxicities were well tolerated with tomotherapy.

In the earlier versions of the tomotherapy machine system, tumor-tracking technology was not available or well equipped. This led to an issue in the intrafractional motion management. Even a non-negligible intrafractional error in a fraction could derail the overall treatment results because of the small fraction numbers.

To tackle this issue, Accuray, which had already clinically implemented the tumor-tracking system for the CyberKnife®, developed a new tracking system called Synchrony® in 2019-2020 for clinical use in the recent tomotherapy machines named Radixact®. With this, the internal and set-up margins can be reduced, like CyberKnife®, in the planning of SBRT for prostate cancer.

Manabe et al. [18] conducted a dosimetric comparison study of 20 patients between CyberKnife® and the Radixact® tomotherapy machine with the newly developed tumor-tracking system. They reported that tomotherapy with the tumor-tracking system had reasonable potential for SBRT for localized prostate cancer as the plans were comparable in terms of maintaining lower doses of the urethra, rectum, and bladder, even though the CyberKnife® system yielded better planning target volume coverage volumes.

Shintani et al. [19] reported their first clinical experience with prostate SBRT using Radixact® with Synchrony® fiducial tracking in their case report.

At our center, out of seven patients who were treated, six patients belonged to the intermediate-risk group (four unfavorable, two favorable) and one patient belonged to the high-risk group according to NCCN. Three patients underwent urological procedures to address the LUTS. The prostate volume prior to treatment ranged from 49 cc to 80 cc. The management protocol for all the patients undergoing SBRT for localized prostate cancer remained largely the same when compared with other studies, except for the omission of hydrogel rectal spacer. HT planning was used in all the cases leading to a conformal and homogenous dose distribution with all the constraints being well within the limits.

No major treatment interruptions due to patient movement or due to excessive rectal gas were encountered, as they were taken up for treatment relatively early in the morning hours while maintaining strict pre-treatment protocols.

The five fractions that were given on consecutive days were well tolerated by the patients, with ≤grade 2 genitourinary toxicity being observed in all at the end of treatment. These toxicities were slightly higher in those who underwent urological procedures to address the LUTS. Following completion, they were continuously followed up for a minimum of five to six months, during which the acute toxicities subsided.

Limitations

There are limitations of the present study. Only few patients who fulfilled the eligibility criteria for undergoing SBRT for prostate cancer were available. Patients with LUTS who have undergone urological procedures may not be good candidates for SBRT, as they might cause an increased incidence of higher grade genitourinary toxicity. Since the Synchrony® system was developed and deployed for clinical use in the recent years and our department acquiring it recently, very little published data are available for the tracking system to build a model to adjust for intrafraction motion of the prostate <2 mm. No definitive statement can be commented upon regarding late toxicities due to the small patient size and short duration of the study. Further studies with a large number of patient cohorts are required.

## Conclusions

Prostate SBRT using Synchrony®-based fiducial tracking shows great promise for intrafraction motion management. By providing real-time tracking, the need for extensive treatment margins is reduced significantly, thereby minimizing radiation exposure to surrounding OARs mainly to the rectum and bladder. Simultaneously, the precision while correcting the offset created due to the bladder and the rectal filling enables improved target conformity and better sparing of normal tissues, potentially leading to better clinical outcomes and reduced toxicity.

Our experience emphasizes the importance of integrating AI-driven tracking systems such as Synchrony® into prostate SBRT for a safer and more effective treatment delivery.
